# High-Speed Mouse Backcrossing Through the Female Germ Line

**DOI:** 10.1371/journal.pone.0166822

**Published:** 2016-12-07

**Authors:** Erin Grove, Sigrid Eckardt, K. John McLaughlin

**Affiliations:** Center for Molecular and Human Genetics, The Research Institute at Nationwide Children’s Hospital, Columbus, Ohio, United States of America; The Roslin Institute, UNITED KINGDOM

## Abstract

Transferring mouse mutations into specific mouse strain backgrounds can be critical for appropriate analysis of phenotypic effects of targeted genomic alterations and quantitative trait loci. Speed congenic breeding strategies incorporating marker-assisted selection of progeny with the highest percentage target background as breeders for the next generation can produce congenic strains within approximately 5 generations. When mating selected donor males to target strain females, this may require more than 1 year, with each generation lasting 10 to 11 weeks including 3 weeks of gestation and 7 to 8 weeks until the males reach sexual maturity. Because ovulation can be induced in female mice as early as 3 weeks of age, superovulation-aided backcrossing of marker-selected females could accelerate the production of congenic animals by approximately 4 weeks per generation, reducing time and cost. Using this approach, we transferred a transgenic strain of undefined genetic background to >99% C57BL/6J within 10 months, with most generations lasting 7 weeks. This involved less than 60 mice in total, with 9 to 18 animals per generation. Our data demonstrate that high-speed backcrossing through the female germline is feasible and practical with small mouse numbers.

## Introduction

Congenic mouse strains are defined as those in which a mutation or genetic alteration has been transferred into a standard inbred mouse strain [1]. Except for the locus of interest and linked chromosomal segment, congenic strains are identical to the selected inbred strain at all genetic loci, minimizing potentially confounding effects of genetic background and reducing experimental variability [2]. Genetic background, i.e. genotypes of all other related genes that may interact with the gene of interest [2], differs between inbred mouse strains and sub-strains, resulting in diverse phenotype manifestations of the same mutation depending on background [3, 4]. For example, profound differences between phenotypic manifestations of double heterozygosity (DH) for targeted deletions of the insulin receptor and insulin receptor substrate-1 genes between C57BL/6 DH (hyperinsulinemia and diabetes) and129S6 DH mice (no diabetes, only mild insulin elevations) have been associated with distinct quantitative trait loci linked to hyperinsulinemia and hyperleptinemia [5].

**Fig 1:**
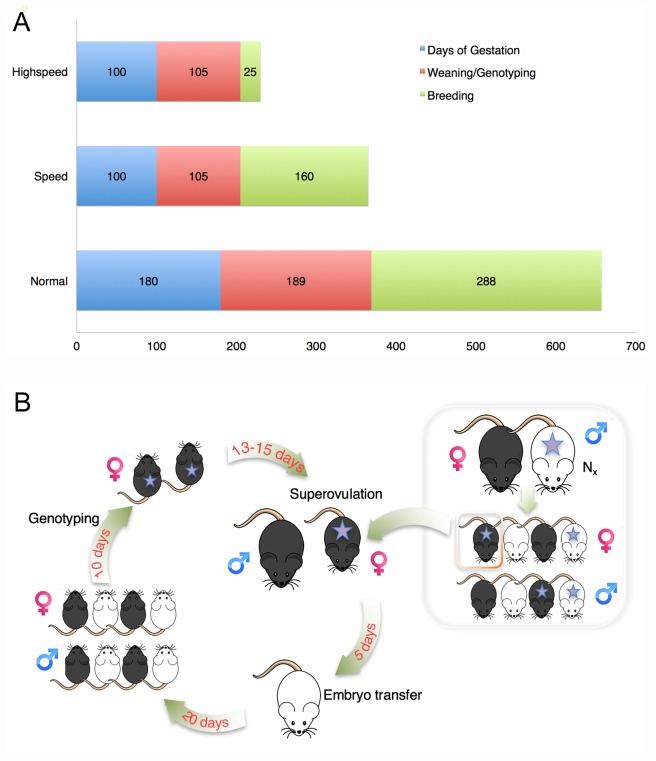
High-speed backcrossing through the female germline. (A) Approximate duration of approaches to obtain congenic strains, based on 5 generations for marker-assisted strategies (high-speed, speed) and 9 generations for traditional backcrossing (normal). Assumed durations: gestation, 20 days; weaning/genotyping, 21 days; breeding, 32 days for speed and normal (assuming maturity of males at 7.5 weeks), and 5 days for high-speed (superovulation at pre-pubertal age). (B) Diagram illustrating the experimental approach for high-speed backcrossing through the female germline. Star, mutation of interest.

Although many types of genetic alterations can now be recreated in specific mouse strains using genome-editing technologies, avoiding the transfer of donor-derived regions flanking the allele of interest, these approaches may not be feasible for large mutations or the exact recapitulation of specific published alleles [6, 7]. Classical congenic breeding entails successive backcrossing to the target strain while selecting for the mutation; theoretically, this results in a congenic strain with 99.9% genetic identity to the target strain after 10 generations [1]. Speed backcrossing incorporates the analysis of single nucleotide polymorphisms (SNPs) distinguishing inbred mouse strains. Selecting progeny with the highest percentage target background as breeders for the next generation can produce congenic strains in about 5 generations [1]. Typically, selected male offspring are mated to target strain females, resulting in a time requirement of about 12 months (50–55 weeks) encompassing 5 generations of 10–11 weeks, including gestation (3 weeks) and sexual maturation of males (7–8 weeks). A suggested but unverified approach to accelerate speed backcrossing combines “supersonic” breeding through the female germline with marker-assisted selection (Fig 1A) [1, 8]. Supersonic breeding relies on superovulation, i.e. the artificially induced ovulation of large numbers of oocytes from female mice using gonadotropin injection. Superovulation is an established approach to increase the yield of oocytes, synchronize experiments involving the use of oocytes or fertilized embryos, and to reduce animal numbers and housing duration [9]. Mouse strains differ in the response to superovulation; however, many commonly used laboratory strains, including C57BL/6J, respond well to superovulation at a pre-pubertal age [10]. When performed with pre-pubertal females, superovulation can therefore accelerate backcrossing by the time period normally required for males to become sexually mature.

Supersonic breeding has performed in the rat [11] but feasibility and outcomes when combined with marker-assisted selection strategies in rodents remain unclear. Here, we investigated the practicality of high-speed backcrossing through the female germline in the laboratory mouse and demonstrate that this approach is feasible and practical with small mouse numbers.

## Materials and Methods

### Animals

This study was carried out in strict accordance with the recommendations in the Guide for the Care and Use of Laboratory Animals of the National Institutes of Health. The protocol was approved by the Animal Care and Use Committee (IACUC) at the Research Institute at Nationwide Children’s Hospital (Permit Number: 02605AR).

C57BL/6J male and female mice were purchased from Jackson Laboratories (Bar Harbor, ME). Animals were either used immediately or bred in brother-to-sister matings for no more than 1 generation to obtain female mice for natural matings. Hsd:ICR (CD-1^®^) recipient females (~8 weeks of age) and males were purchased from Harlan Laboratories (Envigo; Indianapolis, IN). Female CD-1 mice were mated to vasectomized males within 2 months of arrival. All mice were maintained in a SPF barrier facility under a 11:13-h light:dark cycle (lights on, 0700 to 1800) at temperatures of 21 to 24°C. Pathogens excluded from the colony include mouse hepatitis virus, minute virus of mice, NS1 generic parvovirus, mouse parvovirus (MPV1-5), Theiler disease virus, enzootic diarrhea of infant mice, Sendai virus, *Mycoplasma pulmonis*, pneumonia virus of mice, reovirus 3, lymphocytic choriomeningitis virus, mouse adenovirus, and polyoma virus. Non-excluded pathogens are mouse Norovirus, *Helicobacter* sp, *Klebsiella* sp, *Pasteurella* sp and *Proteus* sp. Mice were housed in individually ventilated cages on corncob bedding with ad-libitum access to food (Harlan Global Diet Low Fat Irradiated) and water.

### Superovulation and Embryo Transfer

Female mice were superovulated by intraperitoneal injection of 5 IU pregnant mare’s serum gonadotropin (PMSG, National Hormone & Peptide Program) followed by injection of 5 IU human chorionic gonadotrophin (hCG, Sigma) 48 h later. Females were manually restrained during injections. Females were then mated (1:1) to C57BL/6J stud males, which were maintained in individual cages and ranged in age from 2 to 8 months. Three days after mating, female donors were euthanized by cervical dislocation by a trained investigator with prior IACUC approval for the technique. Blastocyst stage embryos were collected and transferred to the uteri of pseudopregnant Hsd:ICR (CD-1®) recipient females (Harlan Laboratories) using surgical embryo transfer methods as described [9]. Embryo transfers were performed under inhalation anesthesia with isoflurane and all efforts were made to minimize suffering including appropriate postoperative analgesia (buprenorphine). Briefly, anesthetized recipient CD-1 females were placed in a prone position on a warming plate, and following fur removal and sanitizing the incision area, a 5-8mm cm transversal incision was made to expose the body wall and underlying ovarian fat pad. A 0.3 cm vertical incision was then made, and the ovary and end of the uterine horns were exteriorized from the abdominal cavity. Embryos were transferred into the uteri using glass pipettes. After the oviduct and uteri were moved back into the abdominal cavity, the muscle wall was sutured using absorbent sutures and the skin using wound clips. Animals were kept warm and were observed until conscious and fully retaining sternal recumbency.

### Genotyping

In each generation, ear punches from 10-day-old offspring were collected, and genomic DNA was prepared using the HotSHOT method as described [12]. Following genotyping by PCR, DNA samples from transgene-carrying animals were shipped for commercial analysis of mouse strain (target: C57BL/6J) background using a strain-discriminating 384 SNP panel (Charles River Laboratories Max-Bax^®^).

### Guidelines

All sections of this report adhere to the ARRIVE Guidelines for reporting animal research [13]. A completed ARRIVE Guidelines checklist is included in S1 Table.

## Results and Discussion

We investigated the feasibility of “high-speed” backcrossing of marker-selected, super-ovulated pre-pubertal donors (Fig 1A) to generate a congenic C57BL/6J (Jackson Laboratories) strain for a transgenic (tg) strain of undefined background (Fig 1B). In each generation, ear punches from 10-day-old female offspring were PCR-genotyped, followed by immediate shipping of DNA samples from transgene-carrying animals for analysis of target C57BL/6J background using a strain-discriminating 384 SNP panel (Charles River Laboratories Max-Bax®). Results were normally obtained within one week of sample submission such that selected donor females with the highest level of target strain background (C57BL/6J) could be super-ovulated at the age of 21 to 35 days (Table 1), followed by collection of blastocyst stage embryos and their transfer into recipient females (Fig 1B). Repeating this approach with 2 marker-selected females per generation, we obtained mice that were >99% target strain at N5 after 291 days involving a total of 56 mice excluding C57BL/6J stud males and transgene-negative pups sacrificed before weaning (Table 1). The first generation was obtained concomitantly with animal transfer to our facility via shipping of embryos obtained from the mating of 2 founder transgenic males to 5 superovulated C57BL/6J females. We obtained 2 transgene-positive females that had 87.8% and 87.7% target background, indicating that the donor strain had been crossed at least once to C57BL/6J [1]. Except for two generations with unexpected delays (N3 and N5), each subsequent generation took no more than 7 weeks from the date of embryo transfer to the date of the next embryo transfer. In N3, no transgenic females were obtained; instead, available mature transgenic N2 males (n = 2) were mated with mature C57BL/6J females to produce N3 offspring (N3a), adding approximately 30 days. In parallel, we also mated a single N3 transgenic male with a C57BL/6J female and obtained N4 offspring (N4a) within the same time frame as N4 offspring from superovulated N3a females. During N5, obtaining SNP genotyping results was delayed by one month due to relocation of the commercial genotyping laboratory.

**Table 1:** Backcrossing Outcomes.

N	Females superovulated,n	Recipients (embryos transferred) n	Pups (pups kept),n	Tg positive females, n	% C57BL/6J background^a^	Duration of generation, days	Mice used, n^b^
N1	5	2 (10, 12)	2 (2)	2	**87.8/87.6**	47	9
N2	2	2 (10, 10)	9 (2)	3	92.95/**94.13/93.1**	52	10
N3	2	3 (12, 12, 13)	3 (1)	0	n/a	88	18
N3a^c^	n/a	3	24 (6)	2	**97.38/96.6**		
N4	2	2 (10, 11)	9 (2)	2	98.03/**98.43**	49	10
N4a^d^	n/a	1	3 (3)	1	**98.83**	(69)	
N5	2	3 (10, 10, 9)	12 (4)	2 (males)	99.3/99.3	55	9

^a^Animals selected for next generation marked bold

^b^Not including C57BL/6J stud males or tg-negative pups not kept to weaning.

^c^N2 tg-positive males were naturally mated with B6 females.

^d^N3 tg-positive male was naturally mated with B6 female.

Our study shows that high-speed backcrossing through the female germline can reduce the duration of each generation to about 7 weeks, provided that females are genotyped before weaning and strain background genotyping results are obtained within a 7-day window, such that superovulation can be performed in pre-pubertal females. The feasibility of this approach relies critically on a fast turnaround time for these two steps. In the case of major disruptions in the process, such as long delays in genotyping or non-availability of transgenic females in a generation as in our study, male littermates are available to continue breeding. In our example, high-speed backcrossing through the female germline was feasible using a small number of mice. Typically, to obtain congenic mice within 5 generations, screening of 10 to 20 male mice per generation is recommended to allow for optimal selection of animals with highest target strain background; this may require transient expansion of mouse colonies by up to 12 litters, or more than 40 animals per generation [14, 15]. Here, we used 9 to 18 mice per generation (2 selected females, recipients for transfer, and resulting pups). Because the first backcross of an undocumented background resulted in N1 animals with >87% target background, our breeding scheme was presumably accelerated by 1–2 generations [1]. In other cases, more generations may be needed when using such small mouse numbers [14, 15]; however, the shortened duration of each generation would compensate for the total time requirement. Thus, our study shows that speed backcrossing through the female germ line can be used to obtain congenic mice within a comparable frame as speed backcrossing using males but involving fewer animals either for ethical or housing space reasons.

The main difference between traditional and speed backcrossing described here is the use of superovulation, which has several implications including potential effects on genomic imprinting and practicability in specific mouse strains. Because speed backcrossing through the female germline relies on the same approach for marker selection as traditional speed backcrossing, limitations in respect to potentially confounding passenger mutations do not differ between these approaches. Due to the low probability of genetic recombination in regions in close physical proximity to the mutation of interest, donor-derived passenger alleles persist even after extensive backcrossing [16, 17]. These sequences may contain a large number of confounding mutations as suggested by a recent analysis of congenic C57BL/6 genetically modified mouse strains, in which more than 1000 inactivating mutations from the original 129 genome remained present [18].

The use of assisted reproductive techniques, including superovulation and embryo culture, may have effects on genomic imprinting. Several studies have described anomalies in imprinted gene expression when comparing blastocyst stage embryos developing from superovulated versus naturally ovulated oocytes [19–21]; however, subsequent reports found that expression alterations were not associated with abnormalities in methylation patterns [20], and imprinted expression in later stage development was largely normal [22]. Studies investigating postnatal endocrine and reproductive health of offspring resulting from superovulated vs naturally ovulated oocytes found no differences [23, 24]. The question as to whether superovulation would produce confounding effects on the analysis of a particular mutation remains therefore open and is further complicated by the fact that most mouse lines for which congenic breeding strategies are desired, were also derived using targeted mutagenesis approaches that have been implied in potential disruptions of genomic imprinting, including superovulation, embryo culture, and culture of embryonic stem cells, and potential transgenerational effects have not been fully explored [25, 26].

The practicability of high speed backcrossing through the female germline and the number of animals required depends in part on the response to superovulation efficiency, which differs between mouse strains, depends on age and weight, and is additionally influenced by colony management and environmental circumstances [10]. Co-injection of inhibin antiserum and PMS, also termed hyperovulation, is effective in several wild-derived mouse strains and has recently been shown to increase superovulation yields approximately 3-fold in C57BL/6J mice [27]. This method may therefore increase the feasibility of high-speed backcrossing onto a C57BL/6J background and potentially other strains, by providing a larger number of offspring to screen, at the cost of transiently increasing colony size and number of animals involved.

We have demonstrated that superovulation combined with marker-assisted selection of progeny can dramatically reduce the time required for each backcrossing generation. Although using first wave male germ cells from immature mice is potentially even faster [28], it does require advanced technical skills. In summary, we found that speed backcrossing using superovulation of prepubertal females is more labor intensive than conventional speed backcrossing but reduces time, housing and animal husbandry costs. Based on our experience with small animal numbers, future protocols would mandate maintaining two donor locus-positive males from each generation until the next generation has been genotyped. Superovulating any additional donor locus-positive females followed by cryopreservation of the resulting gametes or embryos could also generate experimental redundancy without immediate and potentially unnecessary expansion of animal numbers. Lastly, the feasibility of this method would be greatly enhanced by availability of in-house approaches for SNP genotyping.

## Supporting Information

S1 Table ARRIVE Guidelines Checklist. (PDF)
